# Supplementary material for: Two parallel pathways connect glutamine metabolism and mTORC1 activity to regulate glutamoptosis
Source: Nat Commun. 2021 Aug 10;12:4814. doi: 10.1038/s41467-021-25079-4 (PMC8355106; doi:10.1038/s41467-021-25079-4)
Supplement: Supplementary file 1 — Supplementary Information [file 41467_2021_25079_MOESM1_ESM.pdf]

Two parallel pathways connect glutamine metabolism and mTORC1 activity to regulate glutamoptosis

Clément Bodineau[1,2], Mercedes Tomé[1], Sarah Courtois[3], Ana S.H. Costa[4,5], Marco Sciacovelli[4], Benoit Rousseau[6], Elodie Richard[7], Pierre Vacher[7], Carlos Parejo-Pérez[8], Emilie Bessede[3], Christine Varon[3], Pierre Soubeyran[7], Christian Frezza[4], Piedad del Socorro Murdoch[1,9], Victor H. Villar[10] and Raúl V. Durán[1,2,7,11]

[1] *Centro Andaluz de Biología Molecular y Medicina Regenerativa*, Consejo Superior de Investigaciones Científicas - Universidad de Sevilla - Universidad Pablo de Olavide, Avda. Américo Vespucio 24, 41092 Seville, Spain

[2] *Institut Européen de Chimie et Biologie,* INSERM U1218, Université de Bordeaux, 2 Rue Robert Escarpit, 33607 Pessac, France

[3] *Bordeaux Research in Translational Oncology,* INSERM U1053, Université de Bordeaux, 146 rue Léo Saignat, 33076 Bordeaux cedex, France

[4] *Medical Research Council Cancer Unit,* University of Cambridge, Hutchison/MR Research Centre, Box 197, Cambridge Biomedical Campus, Cambridge CB2 0XZ, UK

[5] Cold Spring Harbor Laboratory, Cold Spring Harbor NY 11724, USA

[6] *Service Commun des Animaleries, Animalerie A2*, University of Bordeaux, Bordeaux, France

[7] *Institut Bergonié,* INSERM U1218, 229 Cours de l'Argonne, 33076 Bordeaux, France

[8] *Instituto de Bioquímica Vegetal y Fotosíntesis*, Consejo Superior de Investigaciones Científicas - Universidad de Sevilla, Avda. Américo Vespucio 49. 41092 Seville, Spain.

[9] *Departamento de Bioquímica Vegetal y Biología Molecular*, Universidad de Sevilla, Avda. Reina Mercedes 6, C.P. 41012, Seville, Spain

[10] CRUK Beatson Institute, Glasgow G61 1BD, UK

[11] To whom correspondence should be addressed: Raúl V. Durán: raul.duran@cabimer.es

Keywords

AMPK, ASNS, glutamine, glutamoptosis, mTORC1, GABA shunt, metabolism

Supplementary Methods

Reagents

A list of the source of the reagents is shown in the Supplementary Table 1.

Supplementary Methods

Supplementary Table 1. Source and usage conditions of all the reagents used in this work.

REAGENT or RESSOURCE	PROVIDER	REFERENCE	DILUTION
**Antibodies**			
rabbit anti-human Phospho-AMPKα (Thr172)	Cell signal	#2535	1:1000
rabbit anti-human AMPKα	Cell Signal	#5832	1:1000
rabbit anti-human Phospho-Raptor (S792)	Cell Signal	#2083	1:1000
rabbit anti-human Raptor (24C12)	Cell Signal	#2280	1:1000
rabbit anti-human Phospho-p70 S6 Kinase (Thr389)	Cell Signal	#9234	1:1000
rabbit anti-human p70 S6 Kinase	Cell Signal	#2708	1:1000
rabbit anti-human Phospho-S6 Ribosomal (Ser235/236)	Cell Signal	#4856	1:1000
rabbit anti-human S6 Ribosomal Protein	Cell Signal	#2217	1:1000
rabbit anti-human Phospho-4E-BP1 (Thr37/46)	Cell Signal	#2855	1:1000
rabbit anti-human 4E-BP1	Cell Signal	#9452	1:1000
rabbit anti-human β-Actin	Cell Signal	#4967	1:1000
rabbit anti-human Glutaminase	abcam	ab93434	1:1000
rabbit anti-human Glutamate Dehydrogenase 1/2	Cell Signal	#12793	1:1000
rabbit anti-human ASNS	Bethyl	A305-331A	1:1000
rabbit anti-human Cleaved Caspase-3 (Asp175)	Cell Signal	#9664	1:1000
rabbit anti-human Cleaved PARP (Asp214)	Cell Signal	#5625	1:1000
mouse anti-human Myc-c-Myc	scbt	sc-40	1:1000
rabbit anti-HA-tag	Sigma Aldrich	SAB4300603	1:1000
goat anti-rabbit HRP-linked	Cell Signal	#7074	1:5000
horse anti-mouse HRP- linked	Cell Signal	#7076	1:5000
mouse anti-CD63	Sigma Aldrich	Sab4700215	1:400
rabbit anti-mTOR	Cell Signal	#2983	1:150
mouse anti-lamp2	abcam	ab25631	1:400
donkey anti-mouse secondary antibody, Alexa Fluor 555	Thermo Fischer	A31570	1:200

donkey anti-rabbit secondary antibody, Alexa Fluor 488	Thermo Fischer	R37118	1:200
**Chemicals**			
BPTES	Sigma Aldrich	SML0601	
DON	Sigma Aldrich	D2141	
DMKG	Sigma Aldrich	349631	
L-Glutamine	Sigma Aldrich	G8540	
L-Leucine	Sigma Aldrich	L8912	
AICAR	Sigma Aldrich	D150959	
Metformin	Sigma Aldrich	PHR1084	
A769662	Santa Cruz Bio.	Sc-203790	
Temsirolimus	Sigma Aldrich	PZ0020	
Oligomycin	Sigma Aldrich	R8875	
FCCP	Sigma Aldrich	C2920	
Rotenone	Sigma Aldrich	R8875	
Antimycin A	Sigma Aldrich	A8674	
**siRNA**			
Control pool:<br>UGGUUUACAUGUCGACUAA<br>UGGUUUACAUGUUGUGUGA<br>UGGUUUACAUGUUUUCUGA<br>UGGUUUACAUGUUUUCCUA	Dharmacon	D-001810	
GLS1 pool:<br>CCUGAAGCAGUUCGAAAUA<br>CGUAAUAUGUGCAUCGAUA<br>AGAAAGUGGAGAUCGAAAU<br>GCACAGACAUGGUUGGUAU	Dharmacon	L-004548	
GDH pool:<br>CCCAAGAACUAUACUGAUA<br>GCGAAGCGCUGUUGCUGUC<br>GAAGAUCUAUGGUUGACUA<br>CCCAUGAAGUGCUAGAUAA	Dharmacon	L-004032	
ASNS pool:<br>GGGUAGAGAUACAUAUGGA<br>UAUGUUGGAUGGUGUGUUU<br>GGUGAAAUCUACAACCAUA<br>GUAAAGAAACGUUUGAUGA	Dharmacon	L-009377	
**Plasmids**			
pRK5-HA GST RagB wt	Addgene	#19301	
pRK5-HA GST RagB 99L	Addgene	#19303	
pRK5-HA GST RagD wt	Addgene	#19307	
pRK5-HA GST RagD 77L	Addgene	#19308	

pcDNA3-AMPKa2-CA	Benoit Viollet	Foretz *et al* 2005 *Diabetes*
pcDNA3.1-HA	Addgene	#128034
**qPCR primers**		
ASNS:<br>Fw: CAGCGGGGACCCAATAGTAG<br>Rv: GTGTAGGACGTGAGCAGAAAA		224589113c1
**Stables isotopes**		
[U-^{13}C]glutamine	Cambridge Isotope Laboratories	CLM-1822-H-MPT
[^{15}N2]glutamine	Cambridge Isotope Laboratories	NLM-1328-PK

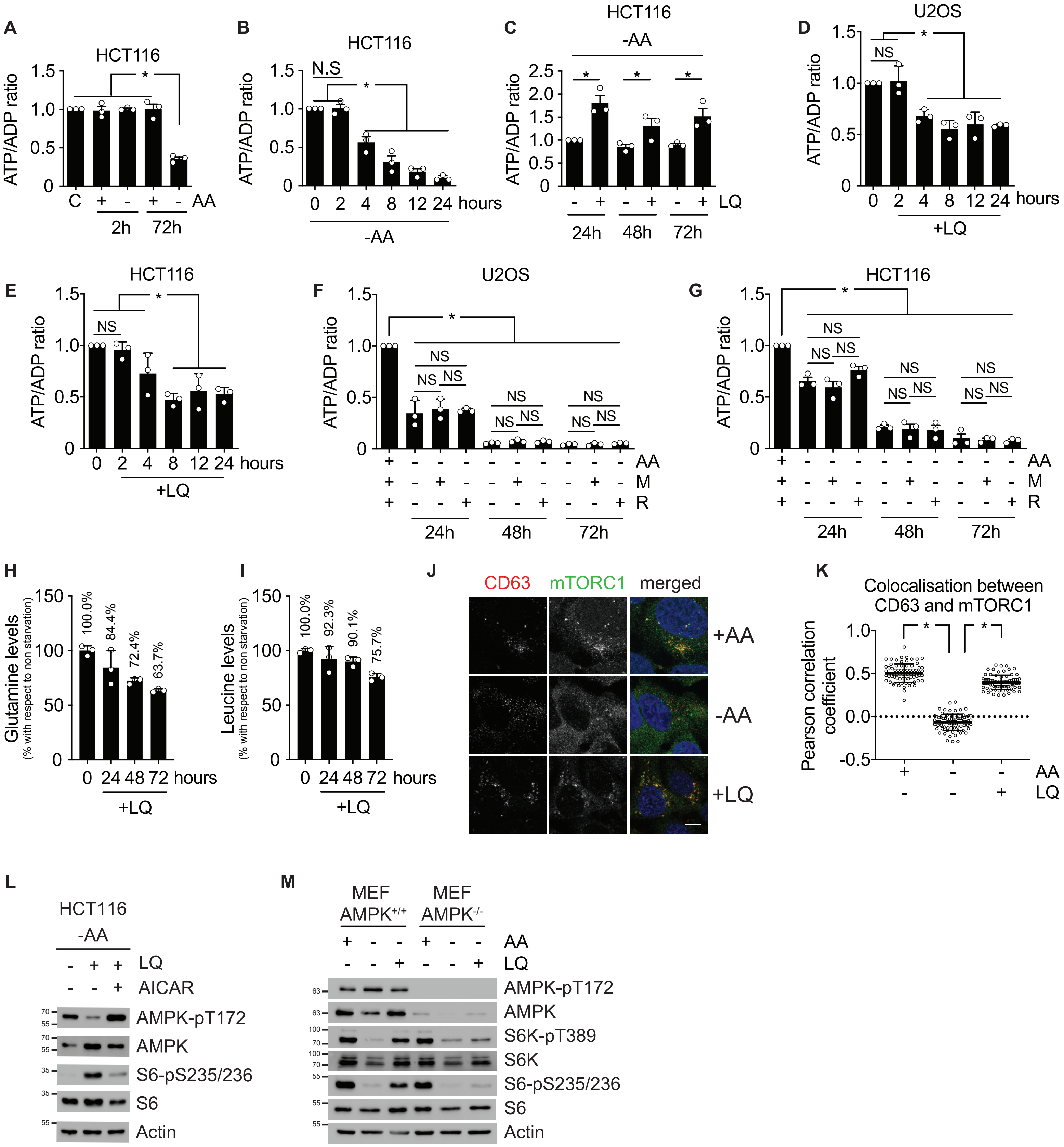
A
HCTT116
ATP/ADP ratio
C + - + - AA
2h 72h
B
HCT116
N.S
0 2 4 8 12 24 hours
-AA
C
HCT116
-AA
- + - + - + LQ
24h 48h 72h
D
U2OS
NS
0 2 4 8 12 24 hours
+LQ
E
HCT116
NS
0 2 4 8 12 24 hours
+LQ
F
U2OS
NS
AA
M
R
24h 48h 72h
G
HCT116
NS
AA
M
R
24h 48h 72h
H
Glutamine levels
(% with respect to non starvation)
100.0%
84.4%
72.4%
63.7%
0 24 48 72 hours
+LQ
I
Leucine levels
(% with respect to non starvation)
100.0%
92.3%
90.1%
75.7%
0 24 48 72 hours
+LQ
J
CD63
mTORC1
merged
+AA
-AA
+LQ
K
Colocalisation between CD63 and mTORC1
Pearson correlation coefficient
+ - - AA
- - + LQ
L
HCT116
-AA
- + + LQ
- - + AICAR
AMPK-pT172
AMPK
S6-pS235/236
S6
Actin
M
MEF AMPK+/+
MEF AMPK-/-
+ - - + - - AA
- - + - - + LQ
AMPK-pT172
AMPK
S6K-pT389
S6K
S6-pS235/236
S6
Actin

Supplementary Figure 1. **Glutaminolysis sustains the production of ATP to inhibit AMPK and to activate mTORC1. (A)** ATP/ADP ratio of HCT116 cells incubated in the presence or the absence of all amino acids for 2 or 72 hours. Fed cells (C) are used as control. **(B)** ATP/ADP ratio of HCT116 cells incubated in the presence or the absence of all amino acids for the indicated time. **(C-E)** ATP/ADP ratio of amino acid-starved HCT116 (C and E) or U2OS (D) cells incubated in the presence or absence of LQ for the indicated times. **(F and G)** ATP/ADP ratio of amino acid-starved U2OS (F) or HCT116 (G) cells incubated in the presence or absence of methionine or arginine for the indicated times. **(H and I)** Remaining levels of glutamine (H) or leucine (I) in the culture medium of U2OS cells incubated for the indicated time with LQ, as estimated by LC-MS analysis. **(J)** Immunofluorescence microscopy captions of U2OS cells incubated with or without amino acids, in the presence or absence of LQ during 72 hours. Cells were stained against CD63 (lysosomal and late endosomal marker, red), mTORC1 (green) and DAPI (blue). Scale bar represents 10µm. **(K)** Quantification of the colocalization between CD63 and mTORC1 as shown in G. Person's R value was evaluated using ImageJ coloc2 plugin on 25 ROI in three biologically independent experiments (75 ROI in total per condition). **(L)** Immunoblot of mTORC1 activity marker (S6 phosphorylation) and AMPK phosphorylation of amino acid-starved HCT116 cells incubated in the presence or absence of LQ, with or without AICAR, during 72 hours. **(M)** Immunoblot of AMPK phosphorylation and mTORC1 activity markers (S6K and S6 phosphorylation) in AMPK $^{+/+}$ or AMPK $^{-/-}$ MEFs incubated in the presence or absence of amino acids or LQ for 72 hours as indicated.

Graphs show mean values ± SEM (n=3 biologically independent experiments). *, $p < 0.05$ (ANOVA analysis followed by a post hoc Bonferroni test). Source data are provided as a Source Data file.

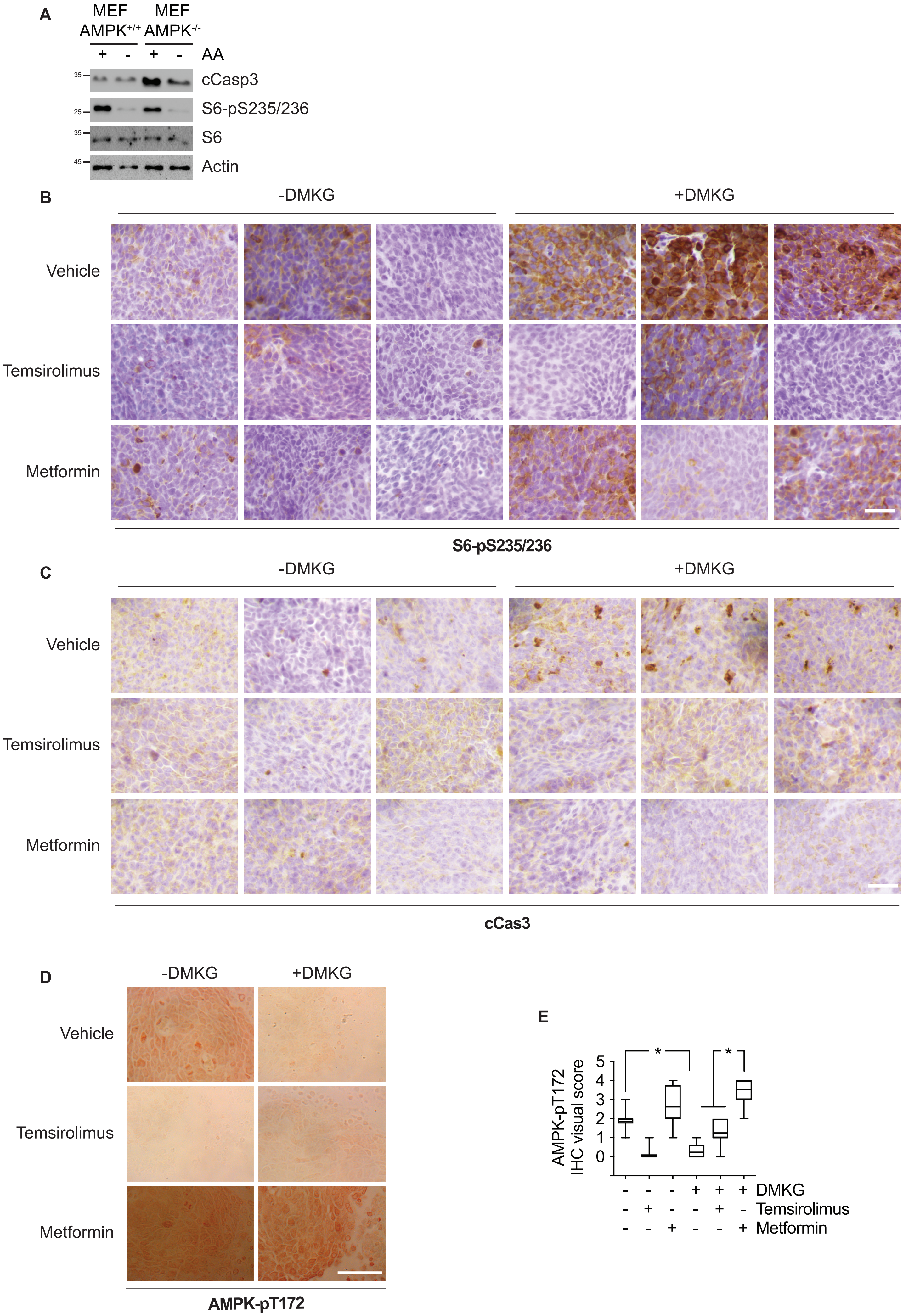
A
MEF AMPK+/+
MEF AMPK-/-
+ - + - AA
35
25
35
45
cCasp3
S6-pS235/236
S6
Actin
B
-DMKG
+DMKG
Vehicle
Temsirolimus
Metformin
S6-pS235/236
C
-DMKG
+DMKG
Vehicle
Temsirolimus
Metformin
cCas3
D
-DMKG
+DMKG
Vehicle
Temsirolimus
Metformin
AMPK-pT172
E
AMPK-pT172
IHC visual score
5
4
3
2
1
0
*
*
- - - + + + DMKG
- + - - + - Temsirolimus
- - + - - + Metformin

Supplementary Figure 2. AMPK inhibition is necessary for glutamoptosis both *in vitro* and *in vivo*. (A) Immunoblot of mTORC1 activity marker (S6 phosphorylation) and apoptotic marker (cleaved caspase 3) in AMPK $^{+/+}$ or AMPK $^{-/-}$ MEFs incubated in the presence or absence of amino acids for 72 hours. **(B** and **C)** Three representative immunohistochemistry microscopy pictures (40X magnification) of xenograft tumors of mice treated as indicated. HCT116 were used to generate xenograft tumors in mice. Samples were stained against S6pS235/236 (B**)** and cleaved caspase 3 (C**)**. **(D)** Representative immunohistochemistry microscopy pictures (40X magnification) of xenograft tumors of mice treated as indicated. HCT116 were used to generate xenograft tumors in mice. Samples were stained against pAMPK-T172. **(E)** Visual score of pAMPK-T172 immunohistochemistry (IHC) images. The upper and lower limits of the boxes represent quartiles, with the line within the boxes indicating the median and the whiskers showing the extremes (n≥10 images per treatment). *, $p < 0.05$ (ANOVA analysis followed by a post hoc Bonferroni test). Source data are provided as a Source Data file.

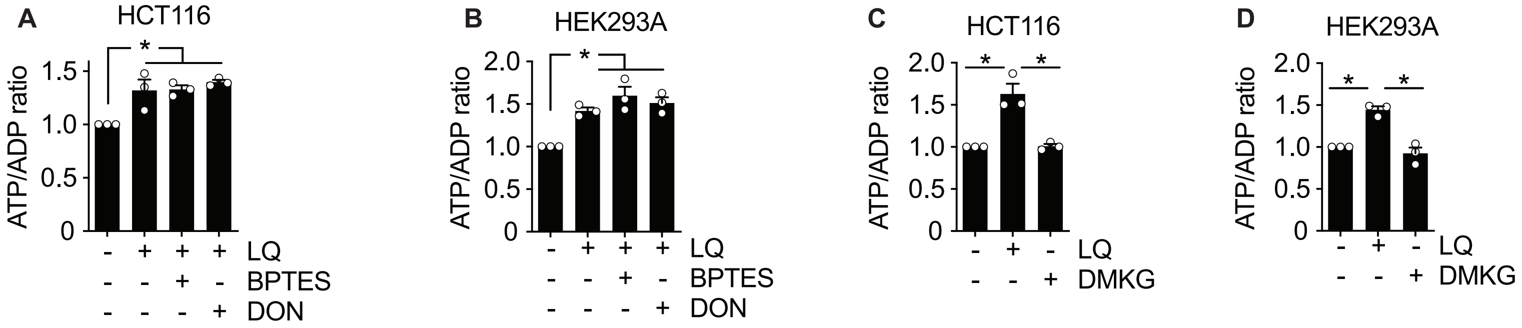
A
HCT116
ATP/ADP ratio
0
0.5
1.0
1.5
*
LQ
BPTES
DON
B
HEK293A
ATP/ADP ratio
0
0.5
1.0
1.5
2.0
*
LQ
BPTES
DON
C
HCT116
ATP/ADP ratio
0
0.5
1.0
1.5
2.0
*
*
LQ
DMKG
D
HEK293A
ATP/ADP ratio
0
0.5
1.0
1.5
2.0
*
*
LQ
DMKG

Supplementary Figure 3. Glutaminolysis is not necessary to induce ATP production. (A and B) ATP/ADP ratio of amino acid-starved HCT116 (A) and HEK293A (B) cells incubated with LQ, BPTES and/or DON for 72 hours. **(C and D)** ATP/ADP ratio of amino acid-starved HCT116 (C) or HEK293A (D) cells with LQ and/or DMKG for 72 hours. Graphs show mean values ± SEM (n=3 biologically independent experiments). *, p < 0.05 (ANOVA analysis followed by a post hoc Bonferroni test). Source data are provided as a Source Data file.

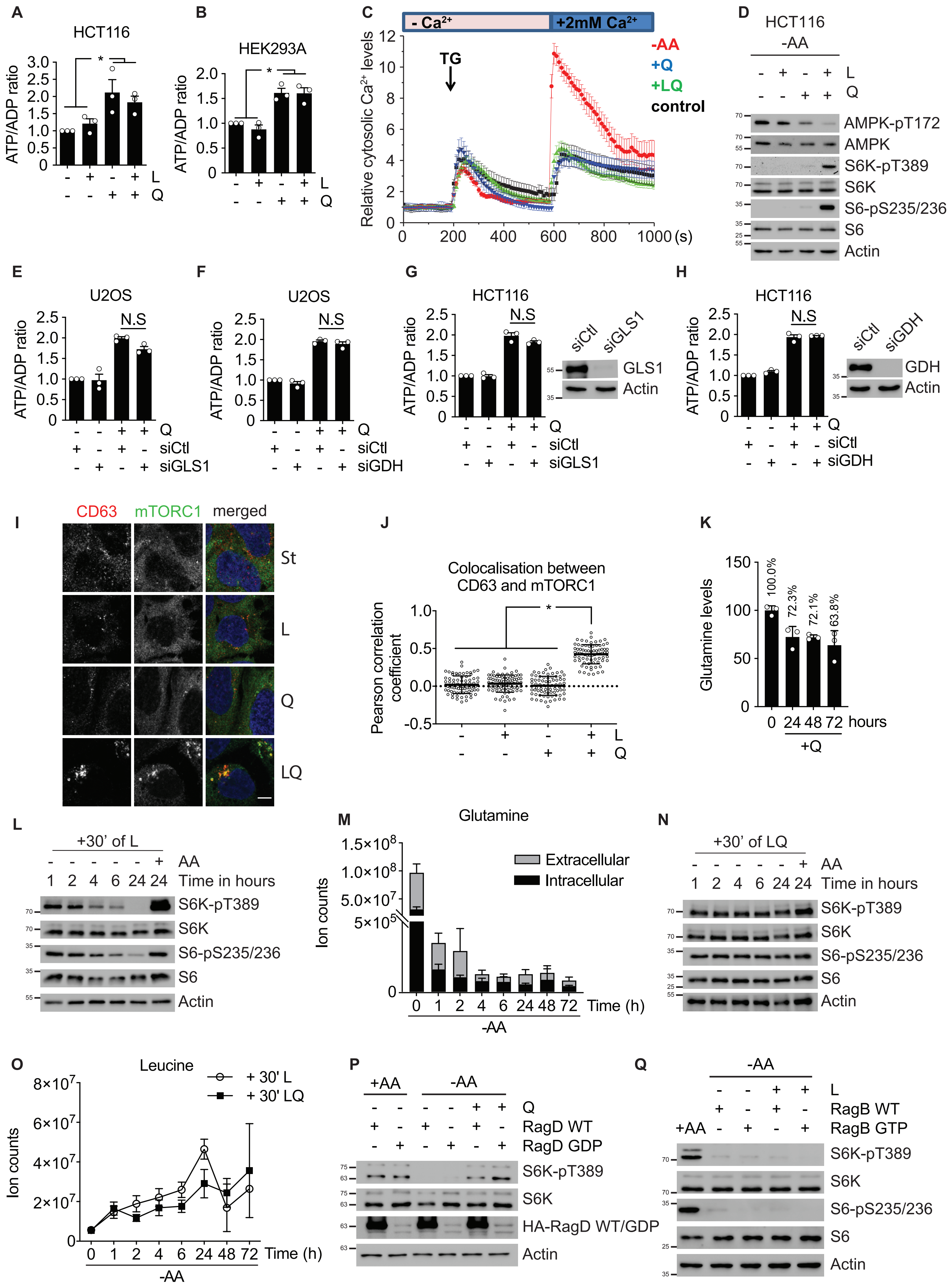
A
HCT116
ATP/ADP ratio
L
Q
B
HEK293A
C
- Ca2+
+2mM Ca2+
TG
-AA
+Q
+LQ
control
Relative cytosolic Ca2+ levels
(s)
D
HCT116
-AA
AMPK-pT172
AMPK
S6K-pT389
S6K
S6-pS235/236
S6
Actin
E
U2OS
N.S
siCtl
siGLS1
F
siGDH
G
HCT116
GLS1
H
GDH
I
CD63
mTORC1
merged
St
LQ
J
Colocalisation between CD63 and mTORC1
Pearson correlation coefficient
K
Glutamine levels
100.0%
72.3%
72.1%
63.8%
hours
+Q
L
+30' of L
AA
Time in hours
M
Glutamine
Extracellular
Intracellular
Ion counts
Time (h)
N
+30' of LQ
O
Leucine
+ 30' L
+ 30' LQ
P
+AA
RagD WT
RagD GDP
HA-RagD WT/GDP
Q
RagB WT
RagB GTP

Supplementary Figure 4. Glutamine metabolism activates mTORC1 following two parallel, necessary branches. **(A** and **B)** ATP/ADP ratio of amino acid-starved HCT116 (A) and HEK293A (B) cells incubated with glutamine and/or leucine. **(C)** U2OS cells were untreated (control) or treated for 48 hours in amino acid starvation with or without Q or LQ and were loaded with the calcium probe Fluo2-LR-AM. Fluorescence intensity changes were normalized to the initial fluorescence value F_0 and expressed as F/F_0 (relative cytosolic calcium levels). After 200 seconds, thapsigargin (1□M) was added to the cells bathed in a Calcium-free medium, in order to estimate ER calcium content. After 600 seconds, calcium was added to the cells to a final concentration of 2mM, in order to estimate capacitive calcium influx. Data represent the mean±SD of F/F0. **(D)** Immunoblot of mTORC1 activity markers (S6K and S6 phosphorylation) and AMPK phosphorylation in amino acid-starved HCT116 cells incubated with glutamine and/or leucine for 72 hours. **(E** and **F)** GLS (E) or GDH (F) expressions were knocked down using small interfering RNA (siRNA) in U2OS cells for 48 hours. Cells were then treated with glutamine for 72 hours and the ATP/ADP ratio was measured. Scramble nontargeting siRNA was used as a control. (**G** and **H**) GLS (G) or GDH (H) expressions were knocked down using small interfering RNA (siRNA) in HCT116 cells for 48 hours. Cells were then treated with glutamine for 72 hours and the ATP/ADP ratio was measured. Scramble nontargeting siRNA was used as a control. Immunoblots of GLS or GDH levels are presented as a control of the knockdown. **(I)** Immunofluorescence microscopy captions of U2OS cells incubated with leucine and/or glutamine during 72 hours. Cells were stained against CD63 (lysosomal and late endosomal marker, red), mTORC1 (green) and DAPI (blue). Scale bar represents 10µm. **(J)** Quantification of the colocalization between CD63 and mTORC1 as shown in K. Person's R value was evaluated using ImageJ coloc2 plugin on 25 ROI in three biologically independent experiments (75 ROI in total per condition). **(K)** Remaining levels of glutamine in the culture medium of U2OS cells incubated for the indicated time with glutamine, as estimated by LC-MS analysis. **(L)** Immunoblot of mTORC1 markers (S6K and S6 phosphorylation) in U2OS cells starved for amino acid for 1, 2, 4, 6 and 24h and restimulated with leucine during 30 minutes. **(M)** Extracellular and intracellular levels of glutamine as determined by LC-MS analysis in U2OS cells upon all amino acid withdrawal for the indicated time. **(N)** Immunoblot of mTORC1 markers (S6K and S6 phosphorylation) in U2OS cells starved for amino acid for 1, 2, 4, 6 and 24h and re-stimulated with leucine and glutamine during 30 minutes. **(O)** Intracellular leucine levels as determined by LC/MS analysis in U2OS cells starved for all amino acids for the indicated time followed by a re-stimulation with either leucine alone or leucine and glutamine during 30 minutes. **(P)** Immunoblot of

mTORC1 marker (S6K phosphorylation) of U2OS cells expressing RagD WT or RagD GDP mutant incubated in the presence or absence of amino acids or glutamine as indicated for 72 hours. **(Q)** Immunoblot of mTORC1 markers (S6K and S6 phosphorylation) of U2OS cells expressing RagB WT or RagB GTP mutant incubated in the presence or absence of amino acids or leucine as indicated for 72 hours. Graphs show mean values ± SEM (n=3 biologically independent experiments). *, $p < 0.05$ (ANOVA analysis followed by a post hoc Bonferroni test). Source data are provided as a Source Data file.

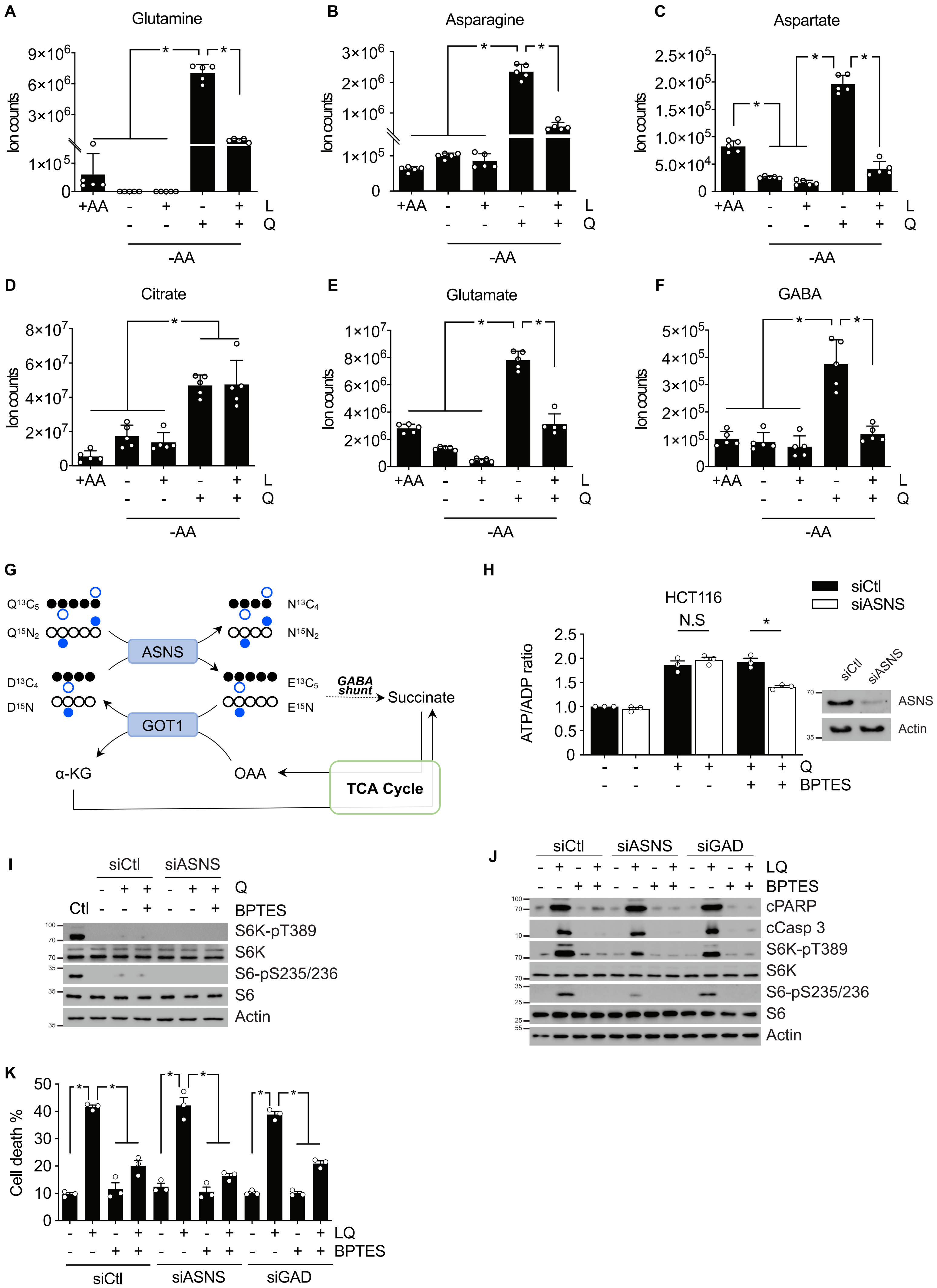
A
Glutamine
Ion counts
9×10^6
6×10^6
3×10^6
1×10^5
0
+AA
L
Q
-AA
B
Asparagine
3×10^6
2×10^6
1×10^6
1×10^5
C
Aspartate
2.5×10^5
2.0×10^5
1.5×10^5
1.0×10^5
5.0×10^4
D
Citrate
8×10^7
6×10^7
4×10^7
2×10^7
E
Glutamate
1×10^7
8×10^6
6×10^6
4×10^6
2×10^6
F
GABA
5×10^5
4×10^5
3×10^5
2×10^5
1×10^5
G
Q13C5
Q15N2
N13C4
N15N2
ASNS
D13C4
D15N
E13C5
E15N
GABA shunt
Succinate
GOT1
α-KG
OAA
TCA Cycle
H
HCT116
siCtl
siASNS
N.S
ATP/ADP ratio
2.5
2.0
1.5
1.0
0.5
0
Q
BPTES
ASNS
Actin
I
siCtl
siASNS
Q
Ctl
BPTES
S6K-pT389
S6K
S6-pS235/236
S6
Actin
J
siCtl
siASNS
siGAD
LQ
BPTES
cPARP
cCasp 3
S6K-pT389
S6K
S6-pS235/236
S6
Actin
K
Cell death %
50
40
30
20
10
0
LQ
BPTES
siCtl
siASNS
siGAD

Supplementary Figure 5. ASNS and GABA shunt are alternative pathways to metabolize glutamine. (A-F) Metabolite levels, as determined by LC-MS analysis, in U2OS cells incubated with or without all amino acids, glutamine and/or leucine as indicated during 72 hours. Total pools of glutamine (A), asparagine (B), aspartate(C), citrate (D), glutamate (E), and GABA (F) are graphed, normalized to protein content. **(G)** Graphical representation describing the metabolism of glutamine by ASNS and the recycling of glutamate through the TCA cycle and GOT1 to produce aspartate. **(H)** ATP/ADP ratio of amino acid-starved HCT116 cells incubated with glutamine and/or BPTES. Immunoblot of ASNS levels is presented as a control of the knockdown. **(I)** Immunoblot of mTORC1 markers (S6K and S6 phosphorylation) of starved U2OS cells upon the silencing of ASNS and in the presence or absence of BPTES and/or glutamine. ASNS expression was knocked down using small interfering RNA (siRNA) during 48 hours. Cells were then treated with glutamine and BPTES as indicated for 72 hours. **(J)** Immunoblot of mTORC1 markers (S6K and S6 phosphorylation) and apoptotic markers (cleaved PARP and cleaved caspase 3) of starved U2OS cells upon the silencing of ASNS or GAD and in the presence or absence of BPTES and/or LQ. ASNS or GAD were knocked down using siRNA for 48 hours and treated with LQ and/or BPTES for 72 hours. **(K)** Cell viability of U2OS cells treated as (J) as determined by trypan blue assay. Graphs show mean values ± SEM (n=3 biologically independent experiments). *, $p < 0.05$ (ANOVA analysis followed by a post hoc Bonferroni test). Source data are provided as a Source Data file.
